# Comparative efficacy and safety of two radiotherapy protocols for ovarian ablation in patients with metastatic breast cancer

**DOI:** 10.3892/mi.2025.255

**Published:** 2025-07-23

**Authors:** Tasneem Hossain, Erika Galietta, Alessio G. Morganti, Abul Farah Md. Kamal Uddin, Shahida Alam, Altaf Hossain, Sonya Begum, Qazi Mushtaq Hussain, Nowshin Taslima Hossain

**Affiliations:** 1 Department of Radiation Oncology, National Institute of Cancer Research and Hospital, Dhaka 1212, Bangladesh; 2 Radiation Oncology, IRCCS Azienda Ospedaliero-Universitaria di Bologna, I-40138 Bologna, Italy; 3 Division of Radiation Oncology, Department of Medical and Surgical Sciences (DIMEC), Alma Mater Studiorum-Bologna University, I-40138 Bologna, Italy; 4 Department of Radiation Oncology, National Institute of ENT, Dhaka 1212, Bangladesh; 5 Department of Radiation Oncology, Khulna Medical College and Hospital, Khulna 9208, Bangladesh; 6 Department of Oncology, Delta Hospital Limited, Dhaka 1216, Bangladesh; 7 Clinical and Radiation Oncology, Labaid Cancer and Superspeciality Centre, Dhaka 1205, Bangladesh; 8 Department of Radiation Oncology, Ahsania Mission Cancer and General Hospital, Dhaka 1230, Bangladesh

**Keywords:** metastatic breast cancer, ovarian ablation, radiotherapy, radiation oncology, breast cancer treatment, hormone receptor positive, radiotherapy dose fractionation, comparative efficacy, treatment toxicity, resource-limited settings

## Abstract

The present study aimed to evaluate the efficacy and safety of radiotherapy (RT) for ovarian ablation (OA) in patients with metastatic breast cancer by comparing two RT protocols: 15 Gy in 5 fractions (arm A) vs. 20 Gy in 10 fractions (arm B). For this purpose, the present study enrolled 68 patients, divided equally into two study arms. The patients were followed-up for 24 weeks post-intervention. The primary endpoint was the efficacy of RT in inducing OA, assessed through amenorrhea and hormone levels [follicle-stimulating hormone (FSH) and estradiol]. Toxicities were evaluated using the Common Toxicity Criteria for Adverse Events version 5.0, and post-menopausal symptoms were assessed using the Menopause Rating Scale. The results revealed that there was no significant difference between the two study arms (A vs. B) in the rate of amenorrhea development and persistence (85.7 vs. 89.5%), and in the achievement of postmenopausal estradiol (91.2 vs. 94.1%) and FSH levels (79.4 vs. 88.2%). Both regimens led to a significant reduction in estradiol levels and an increase in FSH levels compared to baseline levels. No grade ≥3 toxicity was observed. Common postmenopausal symptoms included hot flushes and irritability, with no significant differences between the groups. On the whole, the present study demonstrates that both RT regimens are effective and safe for OA in patients with metastatic breast cancer, with no significant differences in efficacy or toxicity. The findings are particularly relevant in resource-limited settings, underscoring the potential for flexible and shorter treatment regimens in such environment.

## Introduction

Breast cancer (BCa) remains one of the most prevalent tumors affecting women worldwide. According to the World Cancer Research Fund, BCa is the most commonly occurring type of cancer among women and the second most common type of cancer overall, with over two million new cases recorded in 2018(1). In particular, the incidence rate of BCa in Bangladesh according to GLOBOCAN is 105.6 per 100,000 individuals per year (2). This high incidence rate implies the importance of effective treatment modalities.

Ovarian ablation (OA), a critical therapeutic approach, particularly in hormone-receptor-positive metastatic BCa, functions by reducing estrogen production. In fact, estrogen plays a crucial role in the proliferation of hormone-receptor-positive BCa cells. OA can be achieved through several methods: Surgical oophorectomy, radiation-induced OA or pharmacological agents, leading to ovarian suppression. Each method has its distinct mechanisms and implications (3).

Radiotherapy (RT), as a means of OA, has been used for a number of decades due to its non-invasive nature and potential efficacy. RT involves the application of ionizing radiation to the ovarian tissue, leading to follicular destruction and consequently, estrogen deprivation. Moreover, RT is often preferred for OA in low-middle-income countries (LMICs) due to its cost-effectiveness, particularly in comparison to surgical methods or hormonal treatments, such as luteinising hormone-releasing hormone analogs. In fact, RT provides a practical alternative in settings where medical or surgical facilities are limited, reducing the need for postoperative care. Additionally, the simplicity and one-time expense of RT make it more feasible and financially viable for both healthcare systems and patients in resource-constrained environment.

However, the lack of clear guidelines on the optimal dose and fractionation of RT poses challenges. The variability in practice and the absence of a standardized protocol underscore the need for comparative studies (3). Given this background, the present study aimed to compare two different RT protocols for OA in patients with metastatic BCa: A dose of 15 Gy delivered in 5 fractions vs. 20 Gy in 10 fractions. The present comparative analysis is intended to provide clearer insight into the efficacy and safety of these protocols, potentially guiding future clinical practice and standardizing treatment approaches.

## Patients and methods

### Study design and setting

The present study was conducted at the Department of Radiation Oncology, National Institute of Cancer Research and Hospital, Dhaka, Bangladesh, from September 30, 2021 to June 30, 2022. The study aimed to evaluate the efficacy and safety of RT for OA in patients with metastatic BCa. The present study was conducted according to the guidelines of the Declaration of Helsinki and was approved by Ethics Committee of the National Institute of Cancer Research and Hospital, Dhaka, Bangladesh (NICRH/Ethics/2021/280; dated September 30, 2021). Written informed consent was obtained from all subjects involved in the study.

### Patient selection

A total of 68 patients were enrolled in the study, with 34 patients allocated to each study arm by purposive sampling. The inclusion criteria were the following: Premenopausal female patients with a confirmed diagnosis of hormone-receptor-positive, Her-2 negative metastatic BCa, for whom OA was deemed clinically necessary. The exclusion criteria included patients who were in visceral crisis, or who had previous history of ovarian surgery or pelvic RT, or had received chemotherapy within the past 1 year of the study, or those with pre-existing conditions affecting ovarian function.

### Operational definitions

Patients were considered hormone receptor-positive in the present study if, in available immunohistochemical reports performed at another laboratory, they had ≥10% estrogen receptor (ER)- or progesterone receptor (PR)-positive cells. Female patients were deemed to be premenopausal and included in the present study if they had: i) A normal menstrual period within 2 months clinically; or ii) a normal menstrual period within the past 12 months, with serum follicle-stimulating hormone (FSH) and estradiol levels within the premenopausal range. Patients were considered to have developed amenorrhea if they had experienced an absence of menstruation for 3 consecutive months without any subsequent resumption. Serum FSH levels >22 mIU/ml and estradiol levels <30 pg/ml were used as criteria for postmenopausal hormone levels to observe the response. The Common Terminology Criteria for Adverse Events (CTCAE) version 5.0 was used to evaluate radiation-induced toxicities (4). Postmenopausal symptoms were assessed using the standardized Menopause Rating Scale (MRS), which measures health-related quality of life regarding 11 symptoms related to the menopause transition. The presence of a visceral crisis, considered as an exclusion criterion for the study, was defined as extensive visceral metastasis with profound symptomatic involvement, such as lymphangitis carcinomatosis, bone marrow replacement, lung metastases with severe symptoms, carcinomatous meningitis, significant liver metastasis, or a rise in liver function markers to three times above the upper limit.

### RT technique

The RT technique employed was two-dimensional (2D) radiation therapy. For treatment, patients were positioned in a supine position on the treatment table, and radiopaque markers were utilized to outline the pelvic region. Anteroposterior and posteroanterior pelvic fields were designed to fully encompass the ovaries. The delineation of field borders included the following: The inferior border was set at the lower border of the obturator foramen; superiorly, it extended to the inferior sacroiliac joint; and laterally, it was placed 1.5 cm beyond the true pelvic brim. To ensure precise ablation, the localization of the ovaries was verified using ultrasonography. Of note, two different dosing regimens were prescribed for the present study: Arm A received a total dose of 15 Gy, delivered in 5 fractions over the course of 1 week, while arm B was administered 20 Gy in 10 fractions spread over a period of 2 weeks, with treatments administered on consecutive days.

### Assessment of efficacy

The primary endpoint was the efficacy of RT in inducing OA, assessed by the development and persistence of amenorrhea and the attainment of postmenopausal levels of FSH and estradiol. These hormonal levels were measured prior to the commencement of RT and then at 4, 12, and 24 weeks post-RT.

### Statistical analysis

Data were analyzed using appropriate statistical methods to compare the efficacy and safety profiles of the two RT regimens. The primary comparative analysis focused on the rate of OA, while secondary analyses included the assessment of radiation-induced toxicities and menopausal symptoms. The statistical method used was the inferential statistical analysis (Hypothesis testing). Analyses were performed using a two-way mixed ANOVA test followed by the Bonferroni post hoc test and independent t-test for continuous variables and the Chi-squared test and Fisher's exact test for categorical variables. For associations, the Chi-squared test of independence was used. All reported P-values were two-sided and P-values <0.05 were considered to indicate statistically significant differences. As statistical software, IBM SPSS software version 25.0 for windows (IBM SPSS Statistics for Windows, version 25.0; IBM Corp.) was used.

## Results

### Efficacy of RT in OA

**Figure 1:**
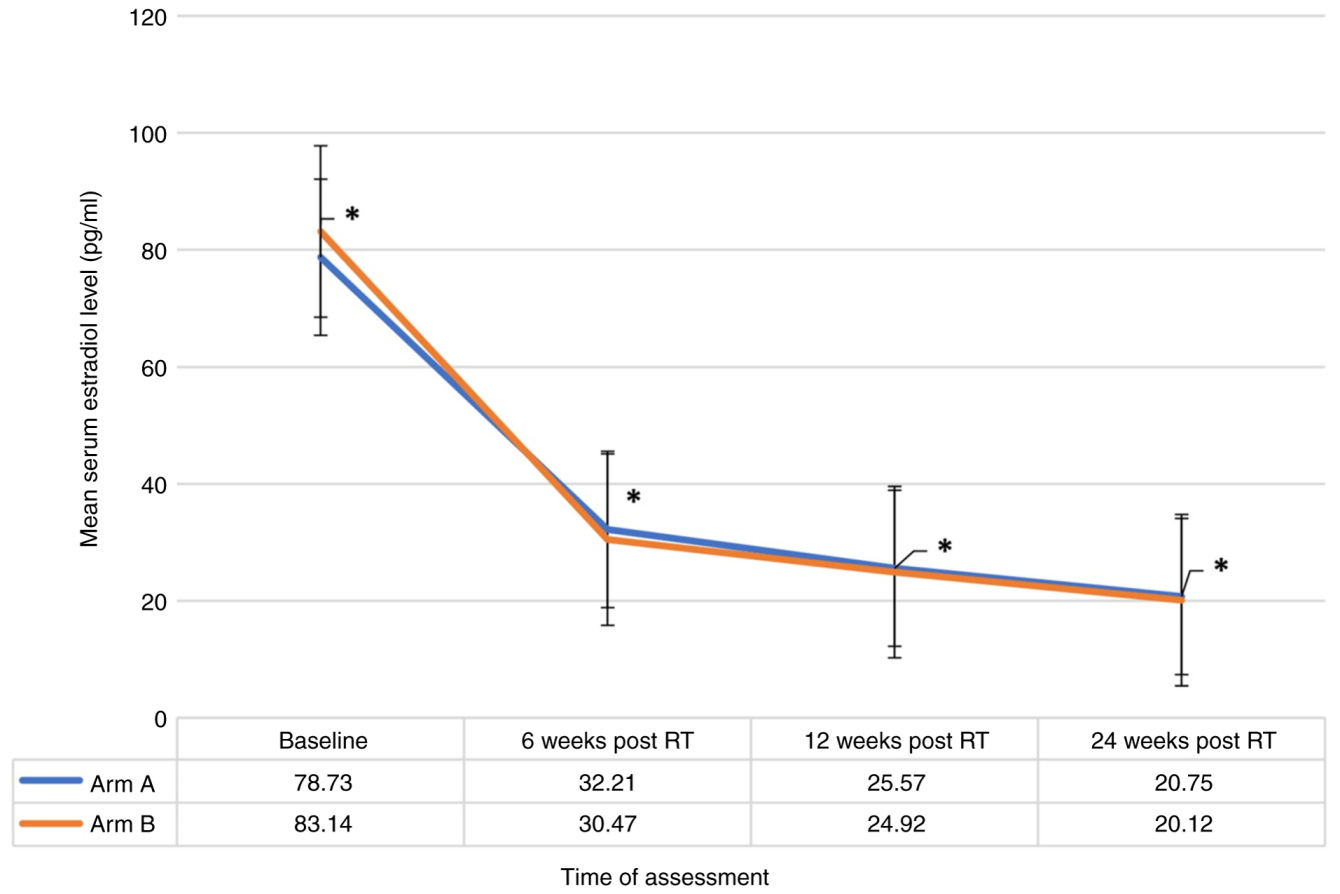
Comparisons of changes in mean serum estradiol levels from baseline during follow-up following radiotherapy in the patients (n=68). The figure illustrates that the estradiol levels consistently decreased over time in both groups. This time-based decrease was statistically significant (^*^P<0.001), as confirmed by the main effect of time (mean value for all patients when compared to previous mean value). Post Hoc pairwise comparisons using the Bonferroni correction confirmed that each successive time point differed significantly from the others (P<0.001), supporting a clear downward trend in estradiol levels over time. However, there was no significant interaction effect between time and group (P=0.133), indicating that the pattern of estradiol decrease over time was similar for both group A and group B. Additionally, there was no strong evidence of a difference in the mean estradiol levels between the two groups (P=0.856). The estradiol levels decreased significantly (P<0.001) and consistently across all four time points. This consistent reduction indicated a progressive hormonal change over the observed periods. Overall, the Bonferroni-adjusted comparisons support that estradiol levels decreased significantly and consistently over time in both groups. RT, radiotherapy.

**Figure 2:**
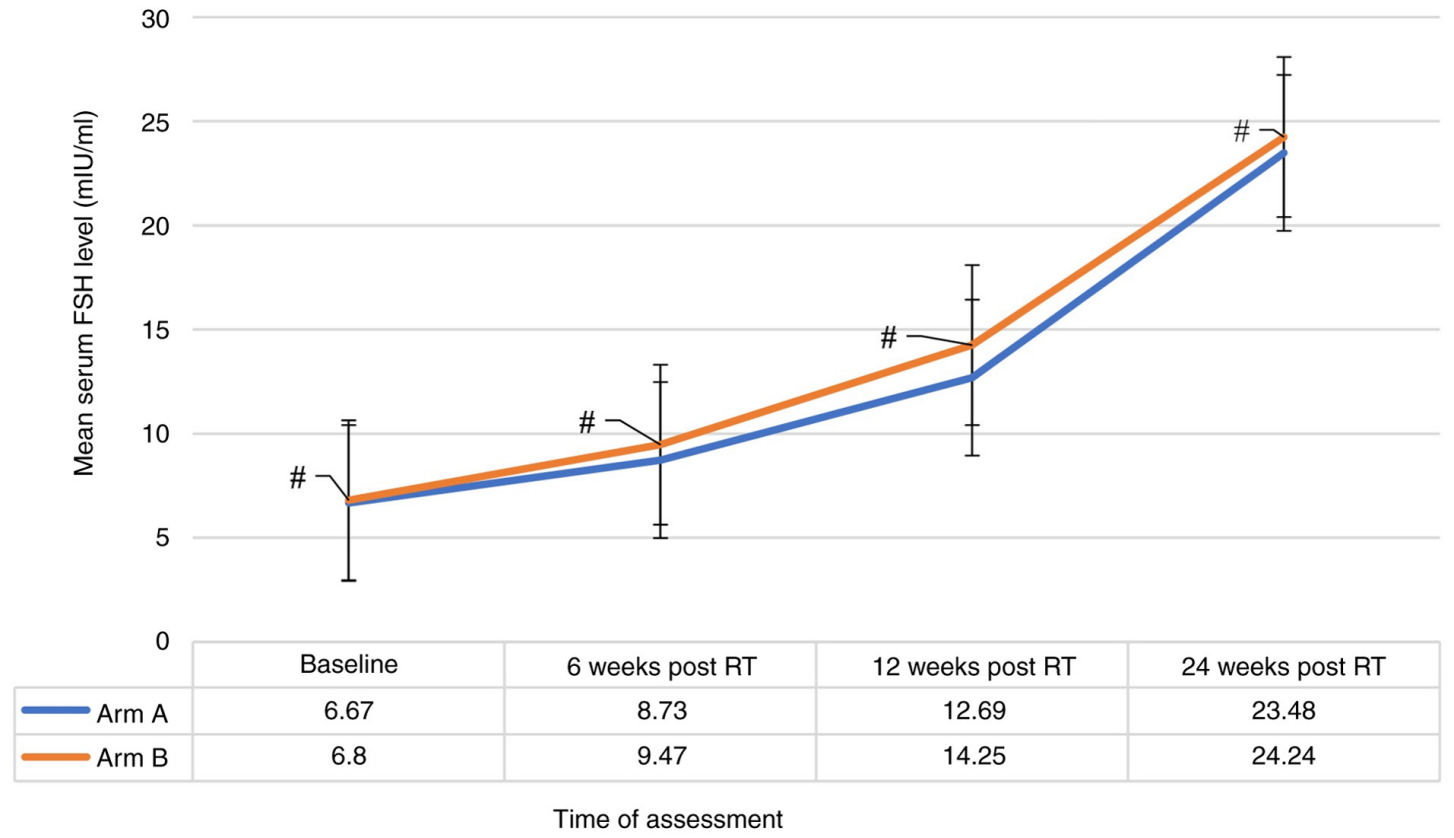
Comparisons of changes in mean serum FSH levels from baseline during follow-up following radiotherapy in the patients (n=68). The figure illustrates that FSH levels consistently increased over time in both groups. This time-based increase was statistically significant (^#^P<0.001), as confirmed by the main effects of time (mean value for all patients when compared to previous mean value). Post hoc pairwise comparisons using the Bonferroni correction confirmed that each successive time point differed significantly from the others (P<0.001), supporting a clear upward trend in FSH level over time. However, there was no significant interaction effect between time and group (P=0.138), indicating the pattern of FSH increase over time was similar for both group A and group B. Additionally, there was no strong evidence of a difference in the mean FSH levels between the two groups (P=0.056). FSH levels increased significantly (P<0.001) and consistently across all four time points. This consistent increase indicated a progressive hormonal change over the observed periods. The Bonferroni-adjusted comparisons support that FSH levels rose significantly and consistently over time in both groups. FSH, follicle-stimulating hormone; RT, radiotherapy.

The patient characteristics are detailed in Table I. In evaluating the efficacy of RT for OA, the present study found no significant differences between arm A (15 Gy in 5 fractions) and arm B (20 Gy in 10 fractions). The rate of development and persistence of amenorrhea was comparable between the two arms, with 85.7% in arm A and 89.5% in arm B (P-value, not significant), excluding 28 (41.17%) patients who had pre-RT amenorrhea with premenopausal hormonal levels. Similarly, the achievement of postmenopausal estradiol levels exhibited no significant differences between the groups, being 91.2% in arm A and 94.1% in arm B (P-value, not significant). The rate of attainment of postmenopausal FSH levels was also similar, with 79.4% in arm A and 88.2% in arm B (P-value, not significant) (Table II). A significant reduction (P-value <0.001) in the mean estradiol level in all the patients included in both groups across all four time points was observed (Fig. 1), alongside a notable consistent significant increase in the mean FSH level at all four points (P-value <0.001) (Fig. 2). However, these changes were not significantly different between the two treatment groups when compared at baseline, 6 weeks, 12 weeks and at 24 weeks indicating that both groups followed a similar pattern of estradiol and FSH levels (Figs. 1 and 2). Additionally, there was no strong evidence of a difference in the mean estradiol (P=0.856) and mean FSH (P=0.056) levels between the two groups.

### Factors influencing efficacy of OA

The analysis revealed that a younger age (<36 years), a higher body mass index (BMI), elevated baseline estradiol levels, and the absence of prior chemotherapy were significantly associated with a failure to achieve OA (P<0.05). These findings suggest that patient-specific characteristics play a crucial role in the response to RT for OA (Table III, Table IV, Table V and Table VI).

### Toxicity and postmenopausal symptoms

As regards safety, the treatment was well tolerated in both arms. No grade ≥3 radiation induced toxicities were observed. The most common postmenopausal symptoms reported by the patients in both study arms were hot flushes and irritability, with no significant difference in their incidence or severity between the two treatment groups (Tables VII and VIII).

## Discussion

The present study aimed to assess the efficacy and safety of RT for OA in patients with metastatic BCa. It primarily investigated the role of RT in inducing OA, assessed through the development and persistence of amenorrhea, and the attainment of postmenopausal levels of FSH and estradiol within 24 weeks of treatment. Using a 2D RT technique, 68 patients were treated with one of two different dose regimens: 15 Gy delivered in 5 fractions (arm A) or 20 Gy delivered in 10 fractions (arm B). The tolerability of these protocols treatments was measured through the CTCAE version 5.0 for radiation-induced toxicities, and the MRS for evaluating post-menopausal symptoms.

The results indicated no significant differences between the two arms in terms of the rate of amenorrhea development, and the achievement of postmenopausal estradiol and FSH levels. In fact, both groups demonstrated a significant decrease in mean estradiol levels and an increase in mean FSH levels compared to baseline, without significant differences between the groups. Furthermore, a younger age, a higher BMI, a high estradiol level, and the absence of prior chemotherapy were significantly associated with the failure to achieve OA. Notably, the study observed no grade ≥3 toxicity, and the most common postmenopausal symptoms were hot flushes and irritability, with no significant differences between the study arms.

The results of the present study can be compared with those of previous studies. In the meta-analysis by Asiri *et al* (5), the efficacy of RT-induced OA was assessed in terms of amenorrhea rates, progression-free survival and overall survival. Their study concluded that RT-OA was effective, with doses of 15 Gy in 5 fractions, 15 Gy in 4 fractions, 16 Gy in 4 fractions, and 20 Gy in 10 fractions demonstrating high amenorrhea rates (5). Comparatively, the present study used doses of 15 Gy in 5 fractions and 20 Gy in 10 fractions, aligning closely with two regimens evaluated in the aforementioned meta-analysis. Both studies found no significant differences in the efficacy of OA between different dose regimens, suggesting that a lower dose may be sufficient for effective OA.

In their retrospective evaluation, Bese *et al* (6) reported a high rate of amenorrhea (96%) with various doses ranging from 5 Gy in a single fraction to 36 Gy in 18 fractions. Their study did not report any severe acute or late complications attributable to RT (6). This aligns with the findings of the present study, where no significant difference in amenorrhea rates was observed between the two study arms, and no severe toxicity was noted. The broad range of doses used in the study by Bese *et al* (6) suggests a potential for flexibility in dosing without compromising the efficacy of OA.

Hughes *et al* (7) reported 75% successful OA using a dose of 20 Gy in 10 fractions, with no reported grade 3 or 4 toxicities. This is consistent with the findings of the present study. In fact, both analyses highlight the relatively low toxicity profile of RT-OA, supporting its safety and tolerability. One of the most notable aspects of the present study is its setting in a LMIC, distinct from the majority of prior studies conducted predominantly in high-income countries (8-12).

In fact, the effectiveness and tolerability of medical therapies, including OA, can be greatly influenced by the treatment setting. This is particularly true in LMICs, where unique challenges, such as the prevalence of specific comorbidities, issues related to malnutrition and the utilization of less advanced medical technologies, including obsolete RT techniques, could potentially affect the outcomes of such treatments. These factors may influence not only the efficacy of the treatment, but also its tolerability, patient compliance and overall outcomes.

Despite these potential limitations, the present study achieved results consistent with those obtained in high-income settings (8-12). This finding is crucial, as it suggests that OA, even when conducted under the constraints typical of a LMIC setting, can be effective and well-tolerated. This is significant, particularly in the context of resource-limited healthcare environments, where access to the latest medical technologies and treatments is often challenging. Moreover, in Bangladesh, radiotherapy-induced ovarian ablation (RT-induced OA) can be performed at a cost as low as $25. This cost is extremely low compared to the $1,200 required for 2 years of ovarian suppression using hormonal agents, or the $600 needed for surgical oophorectomy.

Furthermore, as demonstrated herein, the equivalence in the efficacy of OA administered in 5 sessions, as opposed to 10, holds particular importance in low resource settings. In fact, the scarcity of RT equipment in a number of LMICs often leads to extended waiting lists. Therefore, a shorter treatment regimen not only reduces the burden on healthcare resources, but also improves patient access to timely treatment and compliance to the prescribed treatment. This can be a critical factor in the management of metastatic BCa, where timely intervention can significantly impact patient outcomes and quality of Life.

In conclusion, the findings of the present study not only align with international research (5-7) but also extend its applicability to LMIC contexts. They underscore the potential for adapting and optimizing cancer treatment protocols in resource-limited settings, thereby enhancing the global equity in cancer care.

The present study, while providing key insight into the efficacy of OA using RT, had certain limitations that need to be mentioned. The small sample size and the specific demographic characteristics of the participants may affect the generalizability of the findings to a broader population. Another constraint was the short follow-up period, which limited the authors' ability to assess long-term efficacy in terms of persistence of clinical and hormonal response as well as survival and also late-onset toxicities. Furthermore, being a quasi-experimental study, there may be inherent biases in data collection and analysis methods, potentially affecting the reliability of the results.

On the other hand, the present study also had several strengths. It highlighted the feasibility of a shorter treatment regimen, potentially useful in reducing treatment wait times and improving patient access to care. Moreover, the assessment approach, using both clinical and biochemical markers, provided a comprehensive understanding of the treatment impact. Notably, the safety profile of the treatment regimens using conventional 2D technique was a key finding, with no severe toxicities reported in either treatment arm. This aspect is particularly significant in the settings of metastatic BCa, where patient tolerance and quality of life are paramount.

In summary, while the present study has some limitations typical of quasi-experimental designs, its strengths lie in its practical applicability, comprehensive outcome assessment, and demonstrated safety profile.

## Figures and Tables

**Table I:** Patient characteristics.

Characteristic	Arm A (15 Gy/5 fractions)	Arm B (20 Gy/10 fractions)	All patients
Age (years), median (range)	35 (26-48)	37 (26-49)	36, (26-49)
Previous chemotherapy	30 (88.2%)	29 (85.3%)	59 (86.76%)
Body mass index (kg/m^2^), median (range)	24 (20-32)	23 (20-33)	24 (20-33)

**Table II:** Distribution of patients according to the response to treatment after 24 weeks (n=68).

Treatment response	Arm-A (n=34)		Arm-B (n=34)		P-value
Development of amenorrhea	n=34	%	n=34	%	0.831 (NS)^a^
Prior to RT	13	38.2	15	44.1	
Amenorrhea after RT	18	52.9	17	50.0	
Menstruating after RT	3	8.8	2	5.9	
Estradiol level					0.642 (NS)^a^
Postmenopausal level (<30 pg/ml)	31	91.2	32	94.1	
Premenopausal level (>30 pg/ml)	3	8.8	2	5.9	
FSH level					0.323 (NS)^b^
Postmenopausal level (>22 mIU/ml)	27	79.4	30	88.2	
Premenopausal level (<22 mIU/ml)	7	20.6	4	11.8	

Data were analyzed using

^a^Fisher's exact test or

^b^the Chi-squared test. NS, significant.

**Table III:** Association of amenorrhea with the age of the patients (n=68).

	Amenorrhea						
	Amenorrhea prior to RT (n=28)		After 24 weeks of RT (n=35)		No amenorrhea after 24 weeks of RT (n=5)		
Age group (years)	No. of patients	%	No. of patients	%	No. of patients	%	P-value
26-30	2	7.1	4	11.4	3	60.0	0.013^a^
31-35	3	10.7	10	28.6	2	40.0	
36-40	12	42.9	9	25.7	0	0	
41-45	10	35.7	8	22.9	0	0	
45-50	1	3.6	4	11.4	0	0	

RT, radiotherapy.

^a^Data were analyzed using Fisher's exact test.

**Table IV:** Association of post-RT hormone levels with BMI in the patients (n=68).

A, Estradiol					
	Estradiol level				
	<30 pg/ml (n=63)		>30 pg/ml (n=5)		
BMI (kg/m^2^)	n=63	%	n=5	%	P-value
18.5-24.9 (normal)	41	65.1	1	20	0.009^a,b^
25.0-29.9 (overweight)	19	30.2	2	40	
≥30.0 (obese)	3	4.8	2	40	
B, FSH					
	FSH level				
	>22 mIU/ml (n=57)		<22 mIU/ml (n=11)		
BMI (kg/m^2^)	n=57	%	n=11	%	P-value
18.5-24.9 (normal)	37	64.9	5	45.5	0.467 (NS)^a^
25.0-29.9 (overweight)	16	28.1	5	45.5	
≥30.0 (obese)	4	7	1	9.1	

^a^Data were analyzed using Fisher's exact test.

^b^Indicates a statistically significant difference (P<0.05). NS, not significant; FSH, follicle-stimulating hormone; BMI, body mass index.

**Table V:** Association of post-RT hormone levels with a history of systemic therapy.

A, Estradiol					
	Estradiol level				
	<30 pg/ml (n=63)		>30 pg/ml (n=5)		
Systemic therapy	n=63	%	n=5	%	P-value
Previously received chemotherapy					0.001^**^
Yes	57	90.5	2	40	
No	6	9.50	3	60	
Previously received hormone therapy					0.056 (NS)^**^
Yes	40	63.5	1	20	
No	23	36.5	4	80	
B, FSH					
	FSH level				
	>22 mIU/ml (n=57)		<22 mIU/ml (n=11)		
Systemic therapy	n=57	%	n=11	%	P-value
Previously received chemotherapy					0.001^a,b^
Yes	53	93.0	6	54.5	
No	4	7.0	5	45.5	
Previously received hormone therapy					0.076 (NS)^c^
Yes	37	64.9	4	36.4	
No	20	35.1	7	63.6	

^a^Indicates a statistically significant difference (P<0.05).

^b^Data were analyzed using Fisher's exact test ^**^ and

^c^Chi-squared test. FSH, follicle-stimulating hormone; NS, not significant.

**Table VI:** Association of post-RT hormonal levels with mean baseline hormonal level.

A, Estradiol					
	Post-RT estradiol level				
	<30 pg/ml (n=63)		>30 pg/ml (n=5)		
Baseline hormonal level	Mean	± SD	Mean	± SD	P-value
Mean FSH (mIU/ml)	6.62	±2.26	8.2	±2.4	0.141 (NS)
Range (min-max)	4.0	-15.0	5.5	-12.0	
Mean estradiol (pg/ml)	78.095	±23.87	116.8	±35.513	0.001^a^
Range (min-max)	40.0	-150	84.0-	160.0	
B, FSH					
	Post-RT FSH level				
	>22 mIU/ml (n=57)		<22 mIU/ml (n=11)		
	Mean	± SD	Mean	± SD	P-value
Mean FSH (mIU/ml)	6.68	±2.32	7.0	±2.26	0.682 (NS)
Range (min-max)	4.0	-15.0	4.1	-12.0	
Mean estradiol (pg/ml)	78.965	±24.10	91.18	±36.62	0.164 (NS)
Range (min-max)	40.0	-150	49.0-	160.0	

^a^Indicates a statistically significant difference (P<0.05). FSH, follicle-stimulating hormone; NS, not significant.

**Table VII:** Distribution of the patients in the present study according to toxicity following radiotherapy (n=68).

	Arm-A (n=34)		Arm-B (n=34)		
Acute toxicity	n	%	n	%	P-value
Skin toxicity					0.546 (NS)^a^
Grade 1	3	8.8	4	11.8	
Grade 2	0	0.0	1	2.9	
Absent	31	91.2	29	85.3	
Vomiting					0.535 (NS)^a^
Grade 1	8	23.5	10	29.4	
Grade 2	2	5.9	4	11.8	
Absent	24	70.6	20	58.8	
Nausea					0.965 (NS)^a^
Grade 1	10	29.4	11	32.4	
Grade 2	3	8.8	3	8.8	
Absent	21	61.8	20	58.8	
Abdominal pain					0.400 (NS)^b^
Grade 1	8	23.5	10	29.4	
Grade 2	3	8.8	6	17.6	
Absent	23	67.6	18	52.9	
Diarrhea					0.639 (NS)^a^
Grade 1	4	11.8	6	17.6	
Grade 2	1	2.9	2	5.9	
Absent	29	85.3	26	76.5	
Urinary toxicity					0.554 (NS)^a^
Grade 1	4	11.8	5	14.7	
Grade 2	0	0.0	1	2.9	
Absent	30	88.2	28	82.4	

NS, not significant. Data were analyzed using

^a^Fisher's exact test or

^b^the Chi-squared test.

**Table VIII:** Postmenopausal symptoms of the study patients (according menopause rating scale) (n=68).

	Grade					
Postmenopausal symptoms	0	1	2	3	4	P-value^a^
Hot flush/sweating						
Arm-A	9	10	11	4	0	0.941 (NS)
Arm –B	11	9	11	3	0	
Heart discomfort/palpitation						
Arm-A	14	12	8	0	0	0.835 (NS)
Arm-B	15	13	6	0	0	
Irritability						
Arm-A	9	10	10	5	0	0.950 (NS)
Arm-B	8	12	10	4	0	
Vaginal dryness						
Arm-A	16	9	6	3	0	0.994 (NS)
Arm-B	15	10	6	3	0	
Anxiety						
Arm-A	10	9	10	5	0	0.852 (NS)
Arm-B	11	8	12	3	0	
Dyspareunia/sexual problem						
Arm-A	9	16	6	3	0	0.994 (NS)
Arm-B	10	15	6	3	0	
Bladder problem						
Arm-A	25	9	0	0	0	0.500 (NS)
Arm-B	24	10	0	0	0	
Physical & mental exhaustion						
Arm-A	10	9	10	5	0	0.991 (NS)
Arm-B	9	10	10	5	0	
Joint & muscular discomfort						
Arm-A	26	8	0	0	0	0.500 (NS)
Arm-B	25	9	0	0	0	
Insomnia/sleep problem						
Arm-A	9	10	11	4	0	0.960 (NS)
Arm-B	10	11	9	4	0	
Mood change/depressive mood						
Arm-A	15	13	6	0	0	0.835 (NS)
Arm-B	14	12	8	0	0	

^a^Data were analyzed using Fisher's exact test. NS, not significant.

## Data Availability

The data generated in the present study may be requested from the corresponding author.
